# Crystal structure of a 1,1,2,2-tetra­chloro­ethane-solvated hydrazinecarbo­thio­amide compound

**DOI:** 10.1107/S2056989017010830

**Published:** 2017-08-01

**Authors:** Sayed Riyadh, David L. Hughes, Musa A Said

**Affiliations:** a Chemistry Department, Taibah University, PO Box 30002, Code 14177, Al-Madinah Al-Munawarah, Kingdom of Saudi Arabia; b School of Chemistry, University of East Anglia, Norwich NR4 7TJ, UK

**Keywords:** crystal structure, Schiff base, hydrogen bonding, tautomerism

## Abstract

The title compound, [(1-{4-[2-(2,4-di­hydroxy­phen­yl)diazen-1-yl]phen­yl}ethyl­idene)amino]­thio­urea, 1,1,2,2-tetra­chloro­ethane monosolvate, C_15_H_15_N_5_O_2_S·C_2_H_2_Cl_4_, was prepared from 4-(4-acetyl­phenyl­diazendi­yl)resorcinol and thio­semicarbazide and recrystallized from mixed solvents of tetra­chloro­ethane and *n*-hexane. ^1^H NMR and X-ray diffraction data are in support of the thione tautomeric form. The X-ray analysis shows the mol­ecule crystallizes as a zwitterion, with proton transfer from the nominal phenol to the azide group; the N—N bond length is 1.291 (5) Å, and an intra­molecular N—H⋯O hydrogen bond is formed. In the crystal, N—H⋯O, N—H⋯N and O—H⋯S hydrogen bonds connect the mol­ecules into a three-dimensional network. The tetra­chloro­ethane solvent mol­ecules are linked to this network through weak C—H⋯O linkages.

## Abstract 2

The crystal structure of the title compound, which contains azomethine groups, is reported; its keto and enol tautomeric forms are investigated.

## Chemical context   

**Figure 1:**
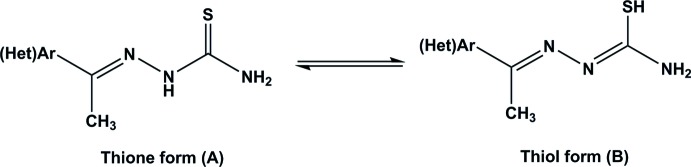
Tautomeric structures of ethyl­idene­thio­semicarbazides.

Ethyl­idene­thio­semicarbazides are polyfunctional compounds with several nucleophilic centers (NH, SH, NH_2_). These compounds exist in both thione and thiol tautomeric forms, Fig. 1 ▸. 1-(1-Aryl­ethyl­idene)thio­semicarbazides have been found to exhibit potent inhibitory activities against mushroom-tyrosinase (a multifunctional copper-containing enzyme that causes dermatological disorders) (Liu *et al.*, 2008 ▸, 2009 ▸). Also, 1-[1-(heterocyclic)ethyl­idene]thio­semicarbazides and their metal complexes have been investigated as potential anti­cancer agents (Finch *et al.*, 2000 ▸; Soares *et al.*, 2012 ▸; Serda *et al.*, 2012 ▸). On the other hand, ethyl­idene­thio­semicarbazides are reactive building blocks for the construction of bioactive heterocycles, such as: [1,2,3]-thia­diazo­les (El-Sadek *et al.*, 2012 ▸], imidazolinones (Thanusu *et al.*, 2010 ▸), thia­zoles (Chimenti *et al.*, 2010 ▸; Abdel-Gawad *et al.*, 2010 ▸; Vazzana *et al.*, 2004 ▸; Vigato & Tamburini, 2004 ▸), and thia­zolidin-4-ones (Abdel-Gawad *et al.*, 2010 ▸). It has been demonstrated that the azomethine group is accountable for biological activities shown by various types of Schiff bases (Vazzana *et al.*, 2004 ▸; Vigato & Tamburini, 2004 ▸). As part of our studies in this area, we now report the synthesis and crystal structure of the solvated title compound, (I), containing azomethine groups and we investigate its keto and enol tautomeric forms.
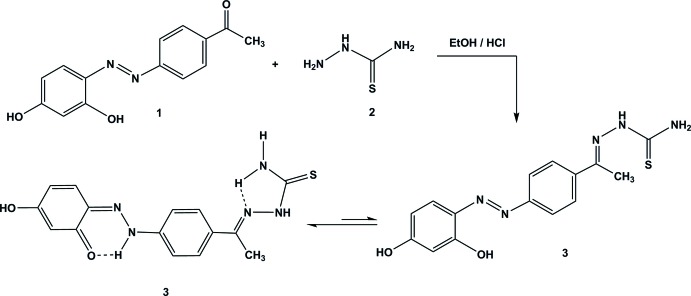


## Structural commentary   

**Figure 2:**
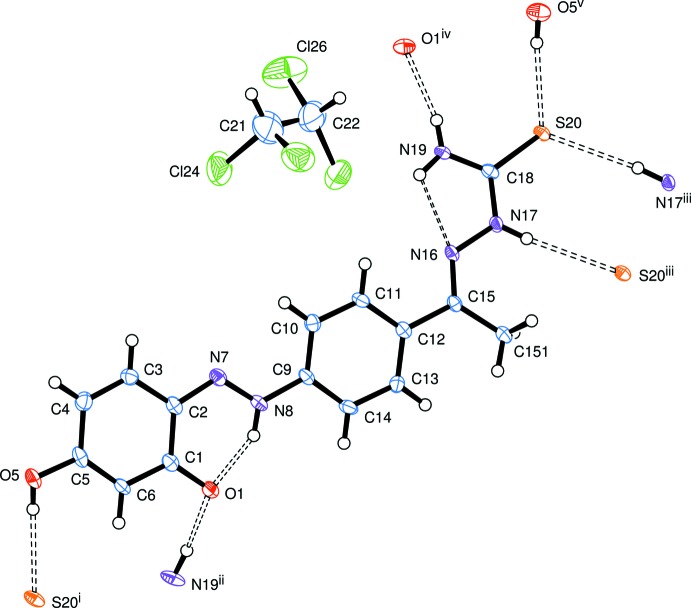
The mol­ecular structure of (I) and the hydrogen-bond inter­actions (not including the ‘weak’ hydrogen bonds). Displacement ellipsoids are drawn at the 50% probability level. Symmetry operations (in the text and all figures): (i) 2 + *x*, 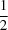 − *y*, *z* − 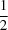; (ii) *x* + 1, 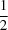 − *y*, 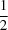 − *z*; (iii) −*x* − 1, 1 − *y*, 1 − *z*; (iv) *x* − 1, 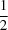 − *y*, 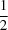 + *z*; (v) *x* − 2, 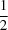 − *y*, 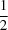 + *z*; (vi) *x* + 1, *y*, *z*; (vii) −*x*, 1 − *y*, 1 − *z*; (viii) *x* − 1, *y*, *z*.

The main mol­ecule comprises two essentially planar groups, which share the C12—C15 bond; the angle between the normals to the two planes is 13.77 (8)°, Fig. 2 ▸.

This mol­ecule is a zwitterion, with a negative charge on O1 and a positive charge on N8: this nitro­gen atom is bonded to a hydrogen atom (clearly identified in the X-ray analysis) and forms an intra­molecular hydrogen bond N8—H8*N*⋯O1. There is delocalized bonding throughout the O1—C1—C2—N7—N8—C9 chain, with bond dimensions very similar to those found in a series of 1-(2-phenyl­diazen-2-ium-1-yl)naphthalen-2-olate compounds studied by Benosmane *et al.* (2013 ▸), Bougueria *et al.* (2013*a* ▸,*b* ▸) and Chetioui *et al.* (2013 ▸), showing a structure midway between the keto and phenolate forms of compound **3** in the Scheme; in particular, the C1—O1 bond length is 1.296 (5) Å and N7—N8 is 1.291 (5) Å.

There is a more pronounced arrangement of double and single bonds for the C=N—N group further along the mol­ecule, with C15=N16 at 1.280 (5) Å, and N16—N17 at 1.377 (5) Å; N16 is the acceptor of a strained hydrogen bond from H19*B*.

**Figure 3:**
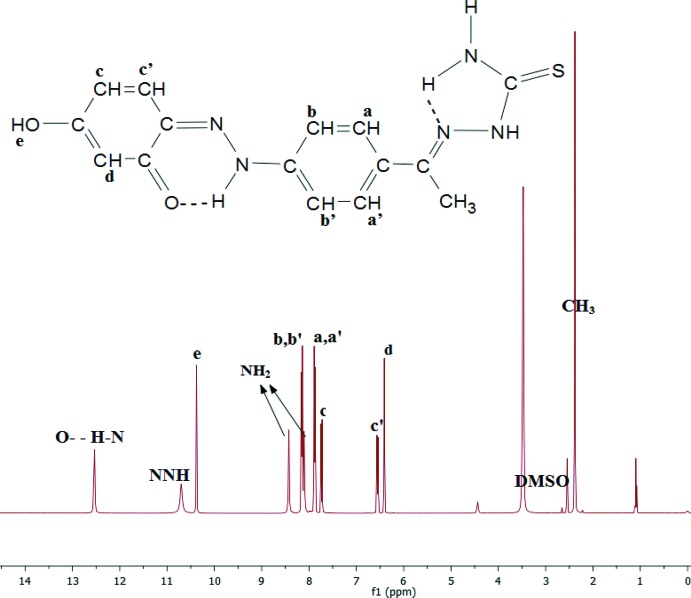
The ^1^H NMR spectrum for compound **3**, showing one form of the product.

The structure of the product was substanti­ated *via* spectroscopic data. For example, ^1^H NMR spectra of compounds **3** revealed two singlet signals at *δ* = 2.35 and 10.28 ppm, attributed to the methyl group adjacent to hydrazone (CH_3_–C=N–NH) (de Oliveira *et al.*, 2014 ▸) and NH of the hydrazone group (C=N–NH), respectively; there are also two signals (*δ* = 8.11 and 8.43 ppm) for the NH_2_ group. A singlet signal at *δ* = 10.70 ppm is due to the OH group whereas the C=O⋯HN appears at *δ* = 12.53 ppm, see Fig. 3 ▸.

## Supra­molecular features   

**Figure 4:**
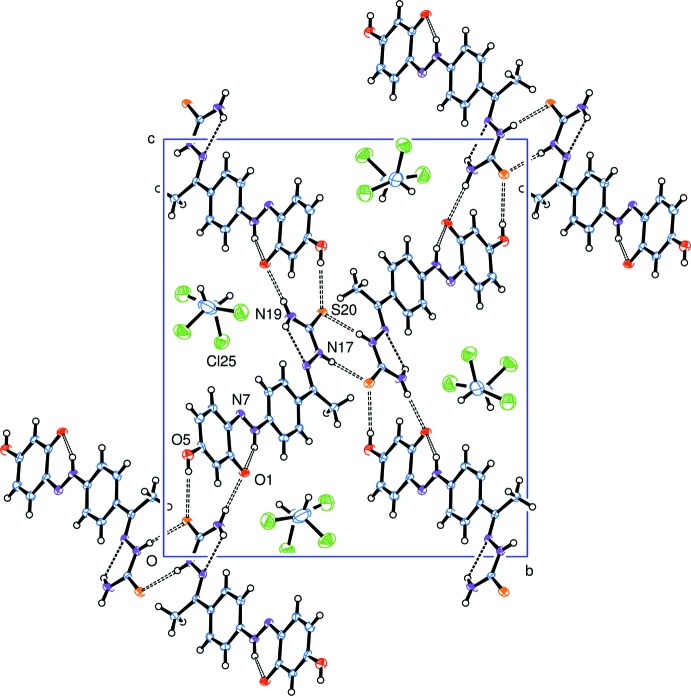
View of the packing in (I) along the *a*-axis direction, showing the hydrogen-bonded system.

**Figure 5:**
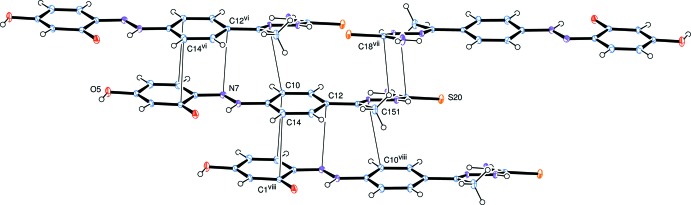
View of the overlapping mol­ecules, showing some of the shorter π–π stacking contacts.

Inter­molecular hydrogen bonds are shown in Table 1 ▸ and Fig. 4 ▸, and connect the mol­ecules into a three-dimensional network. The solvent tetra­chloro­ethane mol­ecules are linked to this network through weak hydrogen bonds C10—H10⋯Cl25^vi^ and C22—H22⋯O1^iv^. Other short inter­molecular contacts connect mol­ecules by π–π stacking along the *a* axis, Fig. 5 ▸; the phenyl ring of C1–C6 lies over the almost parallel ring of C9–C14 in the adjacent mol­ecule, with C1⋯C14^vi^ = 3.309 Å, C3⋯C10^vi^ = 3.360 Å, and C5⋯N8^vi^ = 3.263 Å. The hydrazone group of C15⋯N17 is sandwiched between the C15⋯C18 section of an inverted mol­ecule [with closest contacts of N17⋯N17^vii^ = 3.405 Å and C18⋯H15*C* ^vii^ = 2.83Å] and the chain of N7⋯C11 of the stacked contact [with closest contacts N16⋯C10^viii^ = 3.314 Å and H15*A*⋯C14^viii^ = 2.93 Å].

## Synthesis and crystallization   

4-Acetyl­phenyl­azoresorcinol (Torrey & MacPherson, 1909 ▸) (**1**) (12.8 g, 50 mmol) was dissolved in 100ml of ethanol and stirred with an equimolar qu­antity of thio­semicarbazide (**2**) (4.55 g, 50 mmol) for 24 h at room temperature using catalytic amounts of HCl. The product, precipitated from the reaction mixture, was filtered, washed with ethanol and recrystallized from hot ethanol solution to give compound **3** as dark-red microcrystals (12.34g, 75%). Dark-red prisms of (I) were obtained by recrystallization from mixed solvents of tetra­chloro­methane and *n*-hexane 1:1; m.p. 521–523 K; IR (KBr): ν (cm^−1^) 3456–3257 (OH+NH+NH_2_), 1596 (C=N); ^1^H NMR (DMSO-*d*_6_): *δ* 2.35 (*s*, 3H, CH_3_-C=N-NH), 6.37 (*s*, 1H, =CH—CO), 6.56 (*d*, 1H, *J* = 4 *Hz*, =CH—C=N), 7.69 (*d*, 1H, *J* = 4 *Hz*, =CH—C—OH), 7.90 (*d*, 2H, *J* = 7 *Hz*, Ar-H), 8.25 (*d*, 2H, *J* = 7 *Hz*, Ar-H), 8.11 & 8.50 (2*s*, 2H, NH_2_), 10.28 (*s*, 1H, NH—C=S), 10.75 (*s*, 1H, OH), 12.50 (*s*, 1H, NH—O=C) ppm; ^13^C-NMR (DMSO-*d*_6_): *δ* 13.86 CH_3_—C=N—NH), 102.99 (C6), 109.33 (C3), 121.39 (C10 & C14), 127.64 (C12), 132.54 (C11 & C13), 134.35 (C2), 138.86 (C4), 146.86 (C9), 150.79 (C15), 156.64 (C5), 163.28 (C=O), 179.02 (C=S) ppm; MS *m*/*z* (%): 329 (*M* ^+^, 38), 227 (35), 171 (70), 146 (70). Analysis calculated for C_15_H_15_N_5_O_2_S (329.09): C, 54.70; H, 4.59; N, 21.26; S, 9.74. Found: C, 54.61; H, 4.66; N, 21.33; S, 9.51%.

## Refinement   

Crystal data, data collection and structure refinement details are summarized in Table 2 ▸. The non-hydrogen atoms were refined with anisotropic thermal parameters. Hydrogen atoms on the O and N atoms were located in difference maps and were refined with distance constraints *vi*z O—H distances were set to 0.82 (2) Å and N—H distances to 0.86 Å; their isotropic thermal parameters were refined freely. The remaining H atoms were included in idealized positions with their *U*_iso_ values set to ride on the *U*_eq_ values of the parent carbon atoms.

## Supplementary Material

Crystal structure: contains datablock(s) I. DOI: 10.1107/S2056989017010830/hb7686sup1.cif

Structure factors: contains datablock(s) I. DOI: 10.1107/S2056989017010830/hb7686Isup2.hkl

Supporting information file. DOI: 10.1107/S2056989017010830/hb7686Isup3.cdx

Supporting information file. DOI: 10.1107/S2056989017010830/hb7686Isup4.cml

CCDC reference: 1564085

Additional supporting information:  crystallographic information; 3D view; checkCIF report

## Figures and Tables

**Table 1:** Hydrogen-bond geometry (Å, °)

*D*—H⋯*A*	*D*—H	H⋯*A*	*D*⋯*A*	*D*—H⋯*A*
C6—H6⋯S20^i^	0.93	2.89	3.634 (4)	137
C10—H10⋯Cl25^ii^	0.93	2.88	3.807 (5)	172
C151—H15*C*⋯S20^iii^	0.96	2.87	3.458 (4)	121
N17—H17⋯S20^iii^	0.86	2.63	3.483 (4)	173
C22—H22⋯O1^iv^	0.98	2.37	3.345 (8)	175
O5—H5*O*⋯S20^i^	0.80 (2)	2.43 (2)	3.227 (4)	173 (5)
N8—H8*N*⋯O1	0.85 (2)	1.78 (3)	2.528 (4)	147 (4)
N19—H19*A*⋯O1^iv^	0.84 (2)	2.05 (2)	2.862 (5)	163 (4)
N19—H19*B*⋯N16	0.84 (2)	2.16 (5)	2.578 (5)	111 (4)

Symmetry codes: (i) 
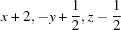
; (ii) 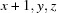; (iii) 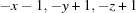; (iv) 
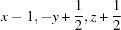
.

**Table 2:** Experimental details

Crystal data	
Chemical formula	C_15_H_15_N_5_O_2_S·C_2_H_2_Cl_4_
*M*_r_	497.21
Crystal system, space group	Monoclinic, *P*2_1_/*c*
Temperature (K)	295
*a*, *b*, *c* (Å)	5.9124 (2), 17.6155 (5), 20.3351 (6)
β (°)	96.563 (3)
*V* (Å^3^)	2104.02 (11)
*Z*	4
Radiation type	Mo *K*α
μ (mm^−1^)	0.69
Crystal size (mm)	0.42 × 0.09 × 0.08

Data collection	
Diffractometer	Oxford Diffraction Xcalibur 3/Sapphire3 CCD
Absorption correction	Multi-scan (*CrysAlis PRO*; Agilent, 2014 ▸)
*T*_min_, *T*_max_	0.728, 1.000
No. of measured, independent and observed [*I* > 2σ(*I*)] reflections	28750, 3675, 3213
*R*_int_	0.053
(sin θ/λ)_max_ (Å^−1^)	0.595

Refinement	
*R*[*F* ^2^ > 2σ(*F* ^2^)], *wR*(*F* ^2^), *S*	0.076, 0.204, 1.09
No. of reflections	3675
No. of parameters	279
No. of restraints	4
H-atom treatment	H atoms treated by a mixture of independent and constrained refinement
Δρ_max_, Δρ_min_ (e Å^−3^)	0.72, −0.79

Computer programs: *CrysAlis PRO* (Agilent, 2014 ▸), *SHELXS97* (Sheldrick, 2008 ▸), *ORTEP* (Johnson, 1976 ▸; Farrugia, 2012 ▸), *SHELXL2014* (Sheldrick, 2015 ▸) and *WinGX* (Farrugia, 2012 ▸).

## supplementary crystallographic information

### Crystal data

**Table:**

C_15_H_15_N_5_O_2_S·C_2_H_2_Cl_4_	*F*(000) = 1016
*M**_r_* = 497.21	*D*_x_ = 1.570 Mg m^−^^3^
Monoclinic, *P*2_1_/*c*	Mo *K*α radiation, λ = 0.71073 Å
*a* = 5.9124 (2) Å	Cell parameters from 5694 reflections
*b* = 17.6155 (5) Å	θ = 3.5–28.7°
*c* = 20.3351 (6) Å	µ = 0.69 mm^−^^1^
β = 96.563 (3)°	*T* = 295 K
*V* = 2104.02 (11) Å^3^	Prism, dark red
*Z* = 4	0.42 × 0.09 × 0.08 mm

### Data collection

**Table:**

Oxford Diffraction Xcalibur 3/Sapphire3 CCD diffractometer	3675 independent reflections
Radiation source: Enhance (Mo) X-ray Source	3213 reflections with *I* > 2σ(*I*)
Graphite monochromator	*R*_int_ = 0.053
Detector resolution: 16.0050 pixels mm^-1^	θ_max_ = 25.0°, θ_min_ = 3.6°
Thin slice φ and ω scans	*h* = −7→7
Absorption correction: multi-scan (CrysAlis PRO; Agilent, 2014)	*k* = −20→20
*T*_min_ = 0.728, *T*_max_ = 1.000	*l* = −24→24
28750 measured reflections	

### Refinement

**Table:**

Refinement on *F*^2^	Primary atom site location: structure-invariant direct methods
Least-squares matrix: full	Secondary atom site location: difference Fourier map
*R*[*F*^2^ > 2σ(*F*^2^)] = 0.076	Hydrogen site location: mixed
*wR*(*F*^2^) = 0.204	H atoms treated by a mixture of independent and constrained refinement
*S* = 1.09	*w* = 1/[σ^2^(*F*_o_^2^) + (0.0938*P*)^2^ + 8.2654*P*] where *P* = (*F*_o_^2^ + 2*F*_c_^2^)/3
3675 reflections	(Δ/σ)_max_ < 0.001
279 parameters	Δρ_max_ = 0.72 e Å^−^^3^
4 restraints	Δρ_min_ = −0.79 e Å^−^^3^

### Special details

**Table:**

Geometry. All esds (except the esd in the dihedral angle between two l.s. planes) are estimated using the full covariance matrix. The cell esds are taken into account individually in the estimation of esds in distances, angles and torsion angles; correlations between esds in cell parameters are only used when they are defined by crystal symmetry. An approximate (isotropic) treatment of cell esds is used for estimating esds involving l.s. planes.

### Fractional atomic coordinates and isotropic or equivalent isotropic displacement parameters (Å^2^)

**Table:**

	*x*	*y*	*z*	*U*_iso_*/*U*_eq_	
O1	0.9543 (5)	0.22114 (17)	0.20187 (14)	0.0225 (7)	
C1	1.0971 (7)	0.1827 (2)	0.2420 (2)	0.0183 (9)	
C2	1.0684 (7)	0.1769 (2)	0.3120 (2)	0.0181 (9)	
C3	1.2286 (7)	0.1342 (2)	0.3548 (2)	0.0217 (9)	
H3	1.2112	0.1308	0.3996	0.026*	
C4	1.4060 (7)	0.0984 (3)	0.3313 (2)	0.0239 (10)	
H4	1.5092	0.0706	0.3596	0.029*	
C5	1.4325 (7)	0.1039 (2)	0.2629 (2)	0.0203 (9)	
O5	1.6154 (5)	0.06701 (19)	0.24376 (17)	0.0270 (7)	
C6	1.2855 (7)	0.1452 (2)	0.2200 (2)	0.0187 (9)	
H6	1.3101	0.1486	0.1757	0.022*	
N7	0.8994 (6)	0.2111 (2)	0.34044 (17)	0.0189 (8)	
N8	0.7553 (6)	0.2510 (2)	0.30244 (17)	0.0184 (8)	
C9	0.5772 (7)	0.2898 (2)	0.3284 (2)	0.0170 (8)	
C10	0.5488 (7)	0.2886 (2)	0.3949 (2)	0.0189 (9)	
H10	0.6486	0.2612	0.4246	0.023*	
C11	0.3698 (7)	0.3286 (2)	0.4168 (2)	0.0194 (9)	
H11	0.3503	0.3277	0.4615	0.023*	
C12	0.2182 (7)	0.3702 (2)	0.3729 (2)	0.0159 (8)	
C13	0.2511 (7)	0.3713 (2)	0.3063 (2)	0.0205 (9)	
H13	0.1541	0.3996	0.2765	0.025*	
C14	0.4281 (7)	0.3304 (3)	0.2839 (2)	0.0221 (9)	
H14	0.4468	0.3303	0.2391	0.027*	
C15	0.0244 (7)	0.4110 (2)	0.3983 (2)	0.0158 (8)	
C151	−0.1144 (7)	0.4677 (3)	0.3562 (2)	0.0235 (10)	
H15A	−0.2643	0.4477	0.3439	0.035*	
H15B	−0.0428	0.4779	0.3171	0.035*	
H15C	−0.1251	0.5139	0.3807	0.035*	
N16	−0.0111 (5)	0.39265 (19)	0.45721 (17)	0.0156 (7)	
N17	−0.1850 (5)	0.42611 (19)	0.48636 (17)	0.0165 (7)	
H17	−0.2660	0.4619	0.4670	0.020*	
C18	−0.2233 (7)	0.4003 (2)	0.5467 (2)	0.0166 (9)	
N19	−0.0835 (7)	0.3482 (2)	0.57381 (19)	0.0249 (9)	
S20	−0.44392 (18)	0.43497 (6)	0.58404 (5)	0.0214 (3)	
C21	0.2745 (18)	0.1205 (5)	0.5978 (4)	0.091 (3)	
H21	0.3296	0.1016	0.6421	0.109*	
C22	0.0344 (16)	0.1387 (4)	0.5960 (4)	0.076 (2)	
H22	0.0155	0.1820	0.6251	0.091*	
Cl23	0.4115 (4)	0.21230 (11)	0.58528 (10)	0.0710 (6)	
Cl24	0.3333 (5)	0.05129 (12)	0.53859 (10)	0.0888 (8)	
Cl25	−0.0877 (4)	0.16005 (11)	0.51483 (9)	0.0729 (6)	
Cl26	−0.0903 (5)	0.05355 (12)	0.62934 (11)	0.0894 (8)	
H5O	1.612 (8)	0.068 (3)	0.2043 (10)	0.019 (13)*	
H8N	0.776 (8)	0.249 (3)	0.2620 (11)	0.021 (12)*	
H19A	−0.101 (7)	0.330 (2)	0.6111 (13)	0.006 (10)*	
H19B	0.023 (6)	0.336 (3)	0.552 (2)	0.025 (13)*	

### Atomic displacement parameters (Å^2^)

**Table:**

	*U*^11^	*U*^22^	*U*^33^	*U*^12^	*U*^13^	*U*^23^
O1	0.0237 (16)	0.0269 (17)	0.0174 (15)	0.0069 (13)	0.0040 (12)	−0.0021 (13)
C1	0.018 (2)	0.016 (2)	0.021 (2)	−0.0031 (16)	0.0043 (17)	−0.0043 (17)
C2	0.016 (2)	0.016 (2)	0.023 (2)	−0.0029 (16)	0.0061 (16)	−0.0041 (17)
C3	0.024 (2)	0.022 (2)	0.021 (2)	0.0003 (17)	0.0072 (18)	0.0012 (17)
C4	0.022 (2)	0.024 (2)	0.026 (2)	0.0029 (18)	0.0021 (18)	0.0023 (18)
C5	0.016 (2)	0.016 (2)	0.029 (2)	−0.0015 (16)	0.0068 (17)	−0.0071 (18)
O5	0.0237 (16)	0.0311 (18)	0.0277 (19)	0.0112 (14)	0.0090 (14)	−0.0021 (15)
C6	0.020 (2)	0.019 (2)	0.018 (2)	0.0012 (17)	0.0079 (17)	−0.0043 (17)
N7	0.0172 (17)	0.0191 (18)	0.0212 (18)	−0.0015 (14)	0.0056 (14)	−0.0021 (15)
N8	0.0173 (18)	0.0221 (19)	0.0167 (19)	0.0001 (14)	0.0065 (14)	−0.0041 (15)
C9	0.0158 (19)	0.017 (2)	0.020 (2)	−0.0013 (16)	0.0074 (16)	−0.0032 (16)
C10	0.017 (2)	0.019 (2)	0.021 (2)	0.0003 (16)	0.0023 (16)	−0.0008 (17)
C11	0.020 (2)	0.024 (2)	0.015 (2)	0.0007 (17)	0.0057 (16)	−0.0029 (17)
C12	0.0152 (19)	0.013 (2)	0.020 (2)	−0.0036 (15)	0.0046 (16)	−0.0029 (16)
C13	0.020 (2)	0.020 (2)	0.022 (2)	0.0050 (17)	0.0049 (17)	0.0043 (17)
C14	0.026 (2)	0.025 (2)	0.017 (2)	0.0006 (18)	0.0104 (18)	−0.0005 (17)
C15	0.0140 (19)	0.014 (2)	0.020 (2)	−0.0013 (15)	0.0043 (16)	−0.0029 (16)
C151	0.024 (2)	0.025 (2)	0.024 (2)	0.0067 (18)	0.0113 (18)	0.0018 (18)
N16	0.0137 (16)	0.0143 (17)	0.0198 (18)	0.0004 (13)	0.0061 (13)	−0.0023 (14)
N17	0.0137 (16)	0.0159 (17)	0.0205 (18)	0.0026 (13)	0.0054 (13)	0.0006 (14)
C18	0.017 (2)	0.016 (2)	0.017 (2)	−0.0047 (16)	0.0062 (16)	−0.0034 (16)
N19	0.028 (2)	0.031 (2)	0.019 (2)	0.0117 (17)	0.0143 (16)	0.0073 (16)
S20	0.0186 (5)	0.0267 (6)	0.0208 (6)	0.0054 (4)	0.0112 (4)	0.0042 (4)
C21	0.134 (8)	0.088 (6)	0.049 (4)	0.040 (6)	−0.003 (5)	−0.025 (4)
C22	0.122 (7)	0.052 (4)	0.053 (4)	0.027 (4)	0.004 (4)	−0.005 (3)
Cl23	0.0926 (14)	0.0562 (11)	0.0691 (12)	−0.0062 (10)	0.0310 (10)	−0.0010 (9)
Cl24	0.136 (2)	0.0733 (13)	0.0533 (11)	0.0549 (13)	−0.0076 (11)	−0.0175 (9)
Cl25	0.1051 (15)	0.0583 (11)	0.0539 (10)	0.0306 (10)	0.0031 (10)	0.0093 (8)
Cl26	0.136 (2)	0.0649 (13)	0.0658 (13)	−0.0453 (13)	0.0050 (12)	0.0031 (10)

### Geometric parameters (Å, º)

**Table:**

O1—C1	1.296 (5)	C12—C15	1.494 (5)
C1—C6	1.412 (6)	C13—C14	1.389 (6)
C1—C2	1.456 (6)	C13—H13	0.9300
C2—N7	1.351 (5)	C14—H14	0.9300
C2—C3	1.426 (6)	C15—N16	1.280 (5)
C3—C4	1.356 (6)	C15—C151	1.499 (6)
C3—H3	0.9300	C151—H15A	0.9600
C4—C5	1.420 (6)	C151—H15B	0.9600
C4—H4	0.9300	C151—H15C	0.9600
C5—O5	1.356 (5)	N16—N17	1.377 (5)
C5—C6	1.369 (6)	N17—C18	1.352 (5)
O5—H5O	0.801 (19)	N17—H17	0.8600
C6—H6	0.9300	C18—N19	1.314 (6)
N7—N8	1.291 (5)	C18—S20	1.696 (4)
N8—C9	1.408 (5)	N19—H19A	0.841 (19)
N8—H8N	0.846 (19)	N19—H19B	0.84 (2)
C9—C10	1.381 (6)	C21—C22	1.451 (13)
C9—C14	1.388 (6)	C21—Cl24	1.776 (8)
C10—C11	1.387 (6)	C21—Cl23	1.840 (11)
C10—H10	0.9300	C21—H21	0.9800
C11—C12	1.398 (6)	C22—Cl25	1.766 (8)
C11—H11	0.9300	C22—Cl26	1.834 (9)
C12—C13	1.392 (6)	C22—H22	0.9800
			
O1—C1—C6	121.7 (4)	C14—C13—H13	119.8
O1—C1—C2	120.8 (4)	C12—C13—H13	119.8
C6—C1—C2	117.5 (4)	C9—C14—C13	120.0 (4)
N7—C2—C3	116.5 (4)	C9—C14—H14	120.0
N7—C2—C1	124.2 (4)	C13—C14—H14	120.0
C3—C2—C1	119.3 (4)	N16—C15—C12	114.6 (4)
C4—C3—C2	121.1 (4)	N16—C15—C151	124.4 (4)
C4—C3—H3	119.4	C12—C15—C151	121.0 (4)
C2—C3—H3	119.4	C15—C151—H15A	109.5
C3—C4—C5	119.3 (4)	C15—C151—H15B	109.5
C3—C4—H4	120.3	H15A—C151—H15B	109.5
C5—C4—H4	120.3	C15—C151—H15C	109.5
O5—C5—C6	122.7 (4)	H15A—C151—H15C	109.5
O5—C5—C4	115.4 (4)	H15B—C151—H15C	109.5
C6—C5—C4	121.8 (4)	C15—N16—N17	120.3 (3)
C5—O5—H5O	110 (4)	C18—N17—N16	117.3 (3)
C5—C6—C1	120.9 (4)	C18—N17—H17	121.4
C5—C6—H6	119.6	N16—N17—H17	121.4
C1—C6—H6	119.6	N19—C18—N17	116.9 (4)
N8—N7—C2	117.1 (4)	N19—C18—S20	122.9 (3)
N7—N8—C9	120.7 (3)	N17—C18—S20	120.2 (3)
N7—N8—H8N	114 (3)	C18—N19—H19A	121 (3)
C9—N8—H8N	125 (3)	C18—N19—H19B	116 (3)
C10—C9—C14	120.5 (4)	H19A—N19—H19B	124 (5)
C10—C9—N8	122.7 (4)	C22—C21—Cl24	113.7 (6)
C14—C9—N8	116.9 (4)	C22—C21—Cl23	104.3 (6)
C9—C10—C11	119.3 (4)	Cl24—C21—Cl23	112.7 (5)
C9—C10—H10	120.4	C22—C21—H21	108.7
C11—C10—H10	120.4	Cl24—C21—H21	108.7
C10—C11—C12	121.2 (4)	Cl23—C21—H21	108.7
C10—C11—H11	119.4	C21—C22—Cl25	111.4 (6)
C12—C11—H11	119.4	C21—C22—Cl26	104.1 (6)
C13—C12—C11	118.6 (4)	Cl25—C22—Cl26	112.4 (5)
C13—C12—C15	121.9 (4)	C21—C22—H22	109.6
C11—C12—C15	119.5 (4)	Cl25—C22—H22	109.6
C14—C13—C12	120.4 (4)	Cl26—C22—H22	109.6
			
O1—C1—C2—N7	−1.3 (6)	C10—C11—C12—C13	−0.5 (6)
C6—C1—C2—N7	178.5 (4)	C10—C11—C12—C15	178.5 (4)
O1—C1—C2—C3	180.0 (4)	C11—C12—C13—C14	1.5 (6)
C6—C1—C2—C3	−0.2 (6)	C15—C12—C13—C14	−177.6 (4)
N7—C2—C3—C4	−179.3 (4)	C10—C9—C14—C13	1.1 (6)
C1—C2—C3—C4	−0.5 (6)	N8—C9—C14—C13	−178.7 (4)
C2—C3—C4—C5	0.2 (7)	C12—C13—C14—C9	−1.7 (6)
C3—C4—C5—O5	179.5 (4)	C13—C12—C15—N16	166.6 (4)
C3—C4—C5—C6	0.9 (7)	C11—C12—C15—N16	−12.5 (5)
O5—C5—C6—C1	180.0 (4)	C13—C12—C15—C151	−12.8 (6)
C4—C5—C6—C1	−1.6 (6)	C11—C12—C15—C151	168.2 (4)
O1—C1—C6—C5	−178.9 (4)	C12—C15—N16—N17	−179.9 (3)
C2—C1—C6—C5	1.2 (6)	C151—C15—N16—N17	−0.6 (6)
C3—C2—N7—N8	178.9 (4)	C15—N16—N17—C18	175.4 (4)
C1—C2—N7—N8	0.2 (6)	N16—N17—C18—N19	3.4 (5)
C2—N7—N8—C9	−178.6 (4)	N16—N17—C18—S20	−176.5 (3)
N7—N8—C9—C10	1.1 (6)	Cl24—C21—C22—Cl25	−50.4 (9)
N7—N8—C9—C14	−179.1 (4)	Cl23—C21—C22—Cl25	72.7 (6)
C14—C9—C10—C11	−0.1 (6)	Cl24—C21—C22—Cl26	71.0 (7)
N8—C9—C10—C11	179.6 (4)	Cl23—C21—C22—Cl26	−165.9 (4)
C9—C10—C11—C12	−0.1 (6)		

### Hydrogen-bond geometry (Å, º)

**Table:**

*D*—H···*A*	*D*—H	H···*A*	*D*···*A*	*D*—H···*A*
C6—H6···S20^i^	0.93	2.89	3.634 (4)	137
C10—H10···Cl25^ii^	0.93	2.88	3.807 (5)	172
C151—H15*C*···S20^iii^	0.96	2.87	3.458 (4)	121
N17—H17···S20^iii^	0.86	2.63	3.483 (4)	173
C22—H22···O1^iv^	0.98	2.37	3.345 (8)	175
O5—H5*O*···S20^i^	0.80 (2)	2.43 (2)	3.227 (4)	173 (5)
N8—H8*N*···O1	0.85 (2)	1.78 (3)	2.528 (4)	147 (4)
N19—H19*A*···O1^iv^	0.84 (2)	2.05 (2)	2.862 (5)	163 (4)
N19—H19*B*···N16	0.84 (2)	2.16 (5)	2.578 (5)	111 (4)

Symmetry codes: (i) *x*+2, −*y*+1/2, *z*−1/2; (ii) *x*+1, *y*, *z*; (iii) −*x*−1, −*y*+1, −*z*+1; (iv) *x*−1, −*y*+1/2, *z*+1/2.

## References

[bb1] Abdel-Gawad, H., Mohamed, H. A., Dawood, K. M. & Badria, F. A.-R. (2010). *Chem. Pharm. Bull.* **58**, 1529–1531. 10.1248/cpb.58.1529 21048349

[bb2] Agilent (2014). *CrysAlis PRO*. Agilent Technologies Ltd, Yarnton, Oxfordshire, England.

[bb3] Benosmane, A., Mili, A., Bouguerria, H. & Bouchoul, A. (2013). *Acta Cryst.* E**69**, o1021. 10.1107/S1600536813014931 PMC3772459 24046602

[bb4] Bougueria, H., Benaouida, M. A., Bouacida, S. & Bouchoul, A. El K. (2013*a*). *Acta Cryst.* E**69**, o1175–o1176. 10.1107/S1600536813017261 PMC3770433 24046718

[bb5] Bougueria, H., Benosmane, A., Benaouida, M. A., Bouchoul, A. El K. & Bouaoud, S. E. (2013*b*). *Acta Cryst.* E**69**, o1052. 10.1107/S1600536813014918 PMC3772484 24046627

[bb6] Cardoso, M. V. de O., de Siqueira, L. R. P., da Silva, E. B., Costa, L. B., Hernandes, M. Z., Rabello, M. M., Ferreira, R. S., da Cruz, L. F., Magalhães Moreira, D. R., Pereira, V. R. A., de Castro, M. C. A. B., Bernhardt, P. V. & Leite, A. C. L. (2014). *Eur. J. Med. Chem.* **86**, 48–59. 10.1016/j.ejmech.2014.08.012 25147146

[bb7] Chetioui, S., Boudraa, I., Bouacida, S., Bouchoul, A. & Bouaoud, S. E. (2013). *Acta Cryst.* E**69**, o1322–o1323. 10.1107/S160053681302014X PMC3793808 24109395

[bb8] Chimenti, F., Bolasco, A., Secci, D., Chimenti, P., Granese, A., Carradori, S., Yáñez, M., Orallo, F., Ortuso, F. & Alcaro, S. (2010). *Bioorg. Med. Chem.* **18**, 5715–5723. 10.1016/j.bmc.2010.06.007 20615716

[bb9] El-Sadek, M. M., Hassan, S. Y., Abdelwahab, H. E. & Yacout, G. A. (2012). *Molecules*, **17**, 8378–8396. 10.3390/molecules17078378 PMC6268412 22785266

[bb10] Farrugia, L. J. (2012). *J. Appl. Cryst.* **45**, 849–854.

[bb11] Finch, R. A., Liu, M.-C., Grill, S. P., Rose, W. C., Loomis, R., Vasquez, K. M., Cheng, Y.-C. & Sartorelli, A. C. (2000). *Biochem. Pharmacol.* **59**, 983–991. 10.1016/s0006-2952(99)00419-0 10692563

[bb23] Johnson, C. K. (1976). *ORTEPII*. Report ORNL-5138. Oak Ridge National Laboratory, Tennessee, USA.

[bb13] Liu, J., Cao, R., Yi, W., Ma, C., Wan, Y., Zhou, B., Ma, L. & Song, H. (2009). *Eur. J. Med. Chem.* **44**, 1773–1778. 10.1016/j.ejmech.2008.04.002 18524420

[bb14] Liu, J., Yi, W., Wan, Y., Ma, L. & Song, H. (2008). *Bioorg. Med. Chem.* **16**, 1096–1102. 10.1016/j.bmc.2007.10.102 18326070

[bb15] Serda, M., Kalinowski, D. S., Mrozek-Wilczkiewicz, A., Musiol, R., Szurko, A., Ratuszna, A., Pantarat, N., Kovacevic, Z., Merlot, A. M., Richardson, D. R. & Polanski, J. (2012). *Bioorg. Med. Chem. Lett.* **22**, 5527–5531. 10.1016/j.bmcl.2012.07.030 22858101

[bb16] Sheldrick, G. M. (2008). *Acta Cryst.* A**64**, 112–122. 10.1107/S0108767307043930 18156677

[bb17] Sheldrick, G. M. (2015). *Acta Cryst.* C**71**, 3–8.

[bb18] Soares, M. A., Lessa, J. A., Mendes, I. C., Da Silva, J. G., Dos Santos, R. G., Salum, L. B., Daghestani, H., Andricopulo, A. D., Day, B. W., Vogt, A., Pesquero, J. L., Rocha, W. R. & Beraldo, H. (2012). *Bioorg. Med. Chem.* **20**, 3396–3409. 10.1016/j.bmc.2012.04.027 22564383

[bb19] Thanusu, J., Kanagarajan, V. & Gopalakrishnan, M. (2010). *Bioorg. Med. Chem. Lett.* **20**, 713–717. 10.1016/j.bmcl.2009.11.074 20004098

[bb20] Torrey, H. A. & MacPherson, W. (1909). *J. Am. Chem. Soc.* **31**, 579–583.

[bb21] Vazzana, I., Terranova, E., Mattioli, F. & Sparatore, F. (2004). *Arkivoc*, (**v**), 364–374.

[bb22] Vigato, P. A. & Tamburini, S. (2004). *Coord. Chem. Rev.* **248**, 1717–2128.

